# PROFILES OF OLDER INCARCERATED PERSONS WHO ATTEMPT SUICIDE FOLLOWING RELEASE

**DOI:** 10.1093/geroni/igad104.1474

**Published:** 2023-12-21

**Authors:** Lisa Barry, Yixia Li, Amy Byers

**Affiliations:** UConn Center on Aging, Farmington, Connecticut, United States; NCIRE, San Francisco, California, United States; UCSF / San Francisco VA Health Care System, San Francisco, California, United States

## Abstract

Release from incarceration is a strong risk factor for suicide. Yet little is known regarding factors associated with suicide attempt among older adults who reentered the community following incarceration. Leveraging a national cohort of U.S. veterans age ≥50 released from incarceration between FY2007 and FY2018 (N=30,584), we conducted a 1:3 matched case-control study and compared demographic and comorbidity factors between those who attempted suicide (cases, N=643) and those with no suicide attempt (controls, N=1,929) up to 13 years post-release. Comorbidity profiles were determined using latent class analysis. As compared with controls, cases had higher proportions of chronic illnesses (e.g., chronic lung disease [35.2% vs. 19.6%], diabetes [32.2% vs. 19.4%], Hepatitis C [24.9% vs. 9.6%]); depression (75% vs. 28.7%); substance use disorders (SUDS) (83.0% vs. 39.4%); and other serious mental illnesses (SMI) (e.g., schizophrenia [34.2% vs. 13.2%], psychotic illness [39.5% vs. 12.5%]). We found four distinct latent clusters among cases (entropy statistic = 0.80): (1) “Minimal Comorbidity” (18%); (2) “PTSD/SUD” (35%); (3) “High Psychiatric/SMI” (32%); and (4) “High Medical/Psychiatric Comorbidity” (15%), and four clusters among controls (entropy statistic = 0.84): (1) “Minimal Comorbidity” (50%); (2) “SMI” (8%); (3) “Tobacco dependence/SUD” (33%); and (4) “High Medical/Psychiatric Comorbidity” (9%). Compared with controls, fewer cases were likely to have the “Minimal Comorbidity” profile (18% vs. 50%), whereas more were likely to have profiles with SMI (32% vs. 8%). Establishing comorbidity profiles of older persons reentering the community after incarceration may help determine who is more likely to die by suicide, thus informing prevention efforts.

